# CLEC5A is critical for dengue virus-induced osteoclast activation and bone homeostasis

**DOI:** 10.1007/s00109-016-1409-0

**Published:** 2016-03-31

**Authors:** Ya-Lang Huang, Szu-Ting Chen, Ren-Shyan Liu, Yen-Hsu Chen, Chun-Yu Lin, Chung-Hao Huang, Pei-Yun Shu, Ching-Len Liao, Shie-Liang Hsieh

**Affiliations:** 1 Institute of Microbiology and Immunology, National Yang-Ming University, Taipei, Taiwan; 2 Department of Microbiology and Immunology, Taipei Medical University, Taipei, Taiwan; 3 Institute of Clinical Medicine, National Yang-Ming University, Taipei, Taiwan; 4 Genomics Research Center, Academia Sinica, 128, Academia Road, Sec. 2, Nankang District, Taipei, 115 Taiwan; 5 Molecular and Genetic Imaging Core, Department of Nuclear Medicine, National Yang-Ming University Medical School and Taipei Veterans General Hospital, Taipei, Taiwan; 6 Division of Infectious Diseases, Department of Internal Medicine, Kaohsiung Medical University Hospital, School of Medicine, College of Medicine, Kaohsiung Medical University, Kaohsiung City, Taiwan; 7 Divisions of Infectious Disease, Center for Disease Control, Taipei, Taiwan; 8 Institute of Infectious Diseases and Vaccinology, National Health Research Institute, Taipei, Taiwan; 9 Department of Medical Research, Taipei Veterans General Hospital, Taipei, Taiwan

**Keywords:** Osteoclasts, Dengue virus, Syk-coupled myeloid C-type lectin member 5A (CLEC5A)

## Abstract

**Abstract:**

Osteoclasts are bone tissue macrophages critical to maintain bone homeostasis. However, whether osteoclasts are susceptible to flaviviral infections and involved in dengue virus (DV)-induced disease pathogenesis is still unknown. In this study, we found that osteoclasts were preferentially susceptible to DV infection and produced similar amounts of cytokines and infectious virions as macrophages. Interestingly, DV-induced cytokine secretion and nuclear translocation of the transcription factor NFATc1 in osteoclast via the Syk-coupled myeloid C-type lectin member 5A (CLEC5A). Moreover, DV caused transient inflammatory reaction in bone tissue and upregulated osteolytic activity to release C-telopeptide of type I collagen (CTX-1) from bone tissue. Furthermore, DV-induced osteolytic activity was attenuated in CLEC5A-deficient mice, and administration of antagonistic anti-CLEC5A mAb inhibited DV-activated osteolytic activity and reduced CTX-1 serum level in vivo. This observation suggests that osteoclasts serve as a novel target for DV, and transient upregulation of osteolytic activity may contribute to the clinical symptoms in dengue patients.

**Key messages:**

- Cultured osteoclasts were susceptible to DV infection.
- Osteoclasts produced similar amounts of cytokines and infectious virions as macrophages.
- DV induced nuclear translocation of NFATc1 in osteoclast via CLEC5A.
- DV caused transient inflammatory reaction in bone tissue and upregulated osteolytic activity.
- Antagonistic anti-CLEC5A mAb inhibited DV-activated osteolytic activity in vivo.

**Electronic supplementary material:**

The online version of this article (doi:10.1007/s00109-016-1409-0) contains supplementary material, which is available to authorized users.

## Introduction

Osteoclasts are multinucleated giant cells differentiated from mononuclear macrophage/monocyte-lineage precursors. Incubation of monocytes with M-CSF and RANK ligand (RANKL) induces monocyte differentiation into osteoclasts in vitro, and stimulation of osteoclast precursors with RANKL in vivo induces cell migration onto the bone surface, where they fuse with each other to form multinucleated giant cells with osteolytic activity. Even though activation of osteoclasts contributes metabolic and inflammatory bone diseases [1, 2], it is still unclear whether osteoclasts are susceptible to viral infections and contribute to immunopathology and disease pathogenesis.

Dengue is one of the most important vector-borne diseases in the world, with approximately 50–100 million cases occurring annually [3]. However, neither effective vaccine nor specific treatment to alleviate clinical symptoms and reduce lethality is available. We have demonstrated that the spleen tyrosine kinase (Syk)-coupled C-type lectin 5A (CLEC5A) interacts with dengue virus (DV) [4] and Japanese encephalitis virus (JEV) [5] directly and is critical for DV-induced hemorrhagic shock and JEV-induced neuroinflammation. Moreover, DV is able to activate NALP3 inflammasome via CLEC5A to secrete IL-1β, IL-18, and other proinflammatory cytokines from GM-CSF-induced inflammatory macrophages [6, 7]. All the evidence suggests that CLEC5A is a critical factor for the pathogenesis of flavivirus-induced systemic inflammatory reactions [8].

In addition, agonistic anti-CLEC5A mAb is able to increase the RANKL-induced osteoclastogenesis in vitro [9], thus enhancing recruitment of inflammatory macrophages and neutrophils to promote bone erosion [10]. The potential role of CLEC5A in the pathogenesis of human rheumatoid arthritis (RA) is further supported by the observation that CLEC5A-positive monocytes increase in active RA patients [11]. All the evidence suggests that CLEC5A is not only critical for flaviviruses-mediated systemic and neuronal inflammation but also involved in regulation of osteoclast activity.

It has been shown that myeloid cells (such dendritic cells and macrophages) are the primary targets for DV infection and replication [4, 12]. Osteoclasts, the tissue macrophages located in bone tissues, are critical to maintain bone homeostasis and bone remodeling. In addition, osteoclasts play a part in pain due to the inflammation adjacent to bone [13], and suppression of osteoclast activity is able to suppress the osteoporotic pain [14]. However, whether osteoclasts are susceptible to DV infection and are involved in the pathogenesis of DV-induced pain sensation has not been investigated yet. To address this question, osteoclasts derived from human monocytes and STAT1-deficient mouse bone marrow were infected with DV2 PL046 strain (DV serotype 2, clinical isolate in Taiwan) for in vitro study, while mouse adapted strain New Guinea C-N strain (DV serotype 2) was used to infect STAT1-deficient mice for in vivo study, as wild-type mice are resistant to dengue virus infection.

Here, we demonstrate that DV not only infects and replicates in osteoclasts but also stimulates the secretion of inflammatory cytokines. Moreover, DV upregulates osteolytic activity via CLEC5A, and CLEC5A-deficient mice are resistant to DV-induced pathological changes. Furthermore, antagonistic anti-CLEC5A mAb inhibits DV-induced osteoclast activation and bone erosion in vivo. Thus, DV-activated osteoclasts may contribute significantly to the clinical symptoms in DV-infected patients, and blockade of CLEC5A has the potential to alleviate DV-induced immunopathology and clinical symptoms in dengue patients.

## Material and methods

### Ethic statement

Human monocytes were obtained from healthy donors at the Taipei Blood Center of the Taiwan Blood Services Foundation, under a protocol (AS-IRB01-14037) approved by the IRB of the Clinical Center of the Department of Health, Taiwan. Written informed consent was obtained from all donors. All animal studies were performed according to the animal study protocol approved by the Animal Experimental Committee of National Yang-Ming University (IACUC #981248) and in accordance with the recommendations in the Guide for the Care and Use of Laboratory Animals of the Taiwanese Council of Agriculture. All surgeries were performed under sodium pentobarbital anesthesia, and every effort was made to minimize suffering.

### Reagents

Culture media/supplements were from Invitrogen GIBCO. Chemical reagents and tartrate-resistant acid phosphatase (TRAP) stain (catalogue no. 387–1) was from Sigma. Macrophage-colony-stimulating factor (M-CSF) and recombinant TNFSF11 (soluble RANKL) for human and mouse, DuoSet ELISA kit for TNF-α, and IL-6 were purchased from R&D Systems. Antibody against NFATc1 (sc-7294) was from Santa Cruz. Bone slice (DT-1BON1000-96), CrossLaps for Culture kit (AC-07F1), RatLaps (AC06F1), and Serum CrossLaps (AC02F1) for CTX-1 detection were purchased from Nordic Bioscience Diagnostics. The PE-conjugated anti-CD51/61 mAb and isotype-matched control for FACS analysis were from BD Pharmingen. Anti-CLEC5A mAb was generated as previous description [4].

### Osteoclast differentiation

The CD14^+^ monocyte-derived osteoclasts were obtained as described previously [15]. For mouse osteoclast differentiation, bone marrow cells (5 × 10^6^) were seeded on Petri dish (10 cm in diameter) for 24 h, followed by collecting the nonadherent cells and incubated in α-MEM medium supplemented with 20 ng/ml of M-CSF and 50 ng/ml of RANKL for further 8 days. Before harvesting, cells were examined under a microscope (Leica Ltd) to determine multinucleated feature and were subjected to the TRAP assay and counterstained with Hoechst/phalloidin to determine osteoclast maturation [16].

### Virus

Dengue virus (DV2-PL046) and New Guinea C-N (DV2) strain were used for all in vitro studies and mouse infection model, respectively, and were propagated in C6/36 mosquito cell line. Viral titers were determined by plaque-forming assays using BHK-21 cells [4]. The neurovirulent Japanese encephalitis virus (RP-9) and the West Nile Virus (B-956 strain, Uganda) were used for comparison with DV in the in vitro assay.

### TRAP staining

Osteoclast formation was determined by detection of TRAP-positive cells as vendor’s instruction (Sigma-Aldrich). Briefly, cells were fixed for 30 s and incubated with a solution containing naphthol AS-BI phosphoric acid at 37 °C for 1 h. The naphthol AS-BI phosphoric acid hydrolyzed by TRAP coupled immediately with fast garnet GBC to form insoluble maroon dye deposited at sites of activity. Stained cells were subjected to a counterstain with a hematoxylin solution. Osteoclasts were determined as TRAP-positive, multinucleated giant cells using light microscopy. The number of TRAP-positive cells and the number of nuclei per TRAP-positive cell in each well were counted, and the morphological features of osteoclasts were also photographed.

### Bone resorption assay

Mature osteoclasts (2 × 10^4^) were seeded on bone slices (Nordic Bioscience Diagnostics) and incubated with DV (M.O.I = 5) at 37 °C in α-MEM media (50 μl/96-well) for 2 h. After removing unbound DV, cells and bone slice were further incubated in fresh α-MEM media (200 μl/96-well). At day 6 postinfection, supernatants were collected and the amount of C-telopeptide of type I collagen (CTX-1) was determined by ELISA kit (CrossLaps for Culture), while bone slices were incubated with 10 % (*v*/*v*) ammonium to remove osteoclast, followed by incubation with 1 % (*v*/*v*) toluidine blue dissolved in 1 % sodium borate for 5 min to detect the resorbed area (pit). Pit numbers of each field were derived from the average of five images per bone slice under inverted microscope (×200).

### Inoculation of virus

Female WT, *Stat1*^*−/−*^ , *Stat*^*−/−*^*Clec5A*^*−/−*^ mice [5] in C57BL/6 background (6–8 weeks old) bread at the YMU Animal Center were inoculated intraperitoneally with 2 × 10^5^ PFUs of DV2 (New Guinea C-N) in 100 μl of PBS, as well as injected intracranially (i.c.) with 30 μl of PBS into the right hemisphere of mouse brains. For in vivo blocking assay, anti-CLEC5A mAb (clone 3D2H6) or isotype control (100 μg per mouse) were administrated intraperitoneally (i.p.) on days 0, 2, 4, and 6 after DV infection.

### Immunofluorescence staining

DV-infected osteoclasts were fixed and permeabilized. Antibody against DV nonstructural protein NS3 (20 μg/ml) or anti-NFATc1 (1:50) were incubated with cells at room temperature for 2 h, followed by incubating with Cy3-conjugated donkey anti-mouse IgG (Jackson ImmunoResearch), then were probed with DyLight 488 Phalloidin (Thermo product no. 21833) at a dilution of 1:500 for 30 min and then counterstained with Hoechst 33342. Cover slips were mounted and observed using an FV-1000 laser scanning microscope.

### SPECT/CT and PET/CT imaging

Each mouse was injected intravenously with a 37 MBq/0.15 ml of ^99m^Tc-methylene diphosphonate (MDP), and the images were acquired at 2 h after injection by using the Triumph^®^ PET/SPECT/CT imaging scanner, preclinical imaging subsystem CZT SPECT, and X-O microCT (TriFoil Imaging, Inc.) for whole-body spiral tomography bone imaging. Sixty-four projections (28 s per projection, ROR 4 cm, FOV 5.28 cm) were made in a 180°, orbit and total acquisition time was 30 min. In PET/CT image, each mouse was injected intravenously with 15–20 MBq/0.1 ml of ^18^F-fluordeoxyglucose (FDG) and the images were acquired at 1 h after injection with the Triumph^®^ PET/SPECT/CT imaging scanner, preclinical imaging subsystem Lab4, and X-O microCT (TriFoil Imaging, Inc.) for inflammatory detection. Before the SPECT and PET scan, the mouse was imaged with CT scan to acquire anatomical information (X-ray source: 50 kV, 0.28 mA; 512 projections). The SPECT and PET image datasets were then reconstructed using the ordered-subset expectation maximization algorithm with standard-mode parameters and 2D maximum likelihood expectation maximization (MLEM) algorithm, respectively. The images were qualitatively interpreted by visual inspection and quantified using AMIDE software (free software provided by SourceForge).

### Imaging acquisition in three-dimensional microcomputed tomography of trabecular bone

The imaging of three-dimensional microcomputed tomography for trabecular bone was collected from each paraformaldehyde-fixed femur by using SkyScan 1076 micro-CT system (Micro Photonics Inc., Belgium). Briefly, data were acquired at 9-μm isotropic voxel size with 360 projections by 180° scan, X-ray voltage of 50 KV, and current of 200 μA. The duration of imaging time was 31 min per scan and followed by 30 min of projection correction and volume reconstruction of three-dimensional representation. Three-dimensional render images of hind paws were generated through original volumetric reconstructed images by CTAn and CTVol software (Micro Photonics Inc.).

### Nuclear-cytoplasmic fractionation

Mature osteoclasts (5 × 10^6^) were seeded on 6 cm dish and incubated with DV (M.O.I = 5) at 37 °C in α-MEM media (1.5 ml/6 cm dish) for 2 h. After removing unbound DV, cells were further incubated in fresh α-MEM media (4 ml/6 cm dish). After 12 h postinfection, cells were harvested for WB analysis. Nuclear-cytoplasmic fractionation was performed using the NE-PER Nuclear and Cytoplasmic Extraction Reagents kit (Thermo Fisher Scientific) according to the manufacturer’s protocol.

### Detection of NFATc1 by immunoblotting

The cytoplasmic (50 μg) and nuclear extracts (15 μg) were separated on 12 % SDS-PAGE before blotting onto PVDF membrane. Lysates were probed with mouse anti-NFATc1 monoclonal antibody (sc-7294 Santa Cruz Biotechnology Inc.) 1:200 in 2.5 % milk/TBST at 4 °C overnight, anti-histone H3 (H0134 sigma) 1:1000 in 2.5 % milk/TBST at 4 °C overnight, or anti-GAPFH (MAB374, Millipore) 1:4000 in 1 % BSA/TBST at room temperature for 1 h. Immunoblots were developed with HRP-conjugated anti-mouse IgG antiserum (cat. no. AP181P; Chemicon) or peroxidase affinipure goat anti-rabbit IgG (H+L) (111-035-144 Jackson Immuno), followed by incubation with SuperSignal™ West Femto Maximum Sensitivity Substrate (34096 Thermo).

### Statistical analysis

All data were presented as mean ± SEM and analyzed using GraphPad Prism software. An unpaired two-tailed Student’s *t* test (for parametric data) or Mann-Whitney test (for nonparametric data) was used to determine the significance between two sets of data. When more than two groups were compared, a one-way ANOVA with the post hoc Bonferroni test (for parametric data) or a Kruskai-Wallis with post hoc Dunn’s test (for nonparametric data) was used for multiple comparisons.

## Results

### Osteoclasts are susceptible to DV infection

**Fig. 1:**
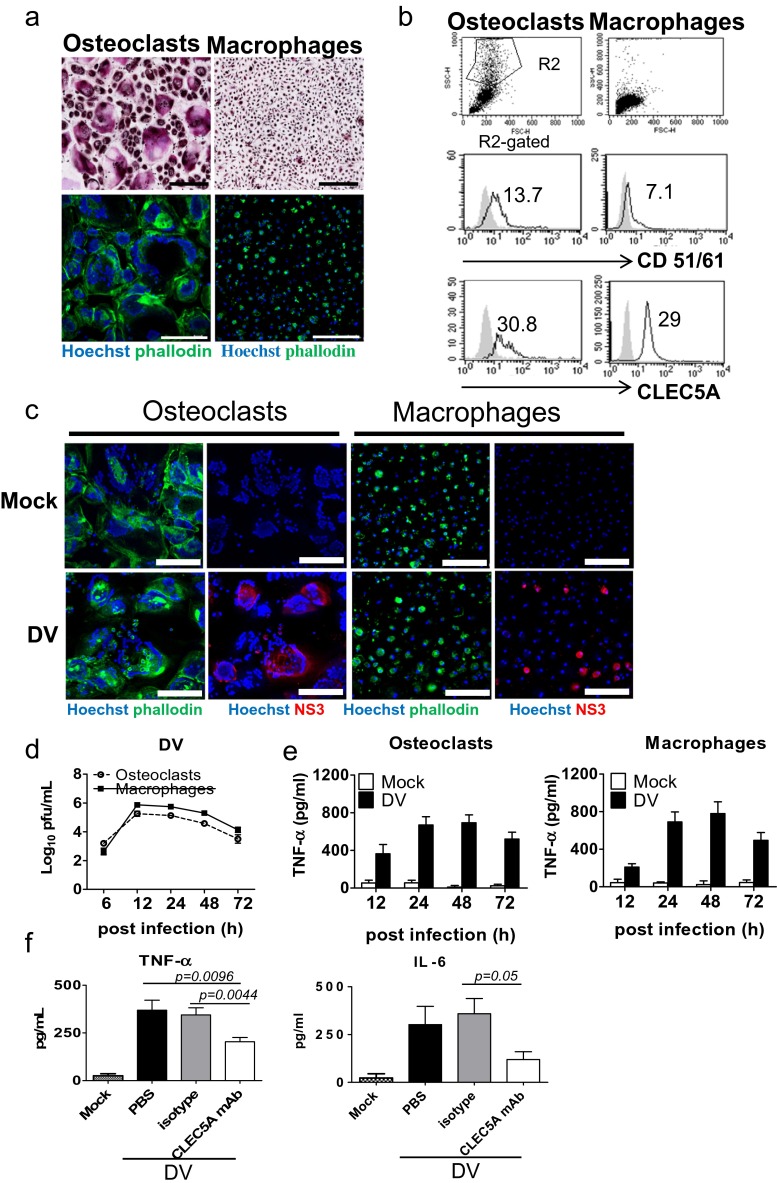
Osteoclasts are susceptible to DV infection and secrete proinflammatory cytokines via CLEC5A. **a** Human osteoclasts (with multinucleated, *left*) and macrophages (*right*) with TRAP (*upper*, *purple red color*) and phalloidin (*lower*, *green color*) staining, respectively. *Scale bar*, 200 μm. **b** Detection of osteoclast markers (CD51/61) and CLEC5A in human osteoclasts and macrophages by flow cytometry (*gray shadow*: isotype control; mean fluorescence intensity of each antibody staining was indicated). **c** Human osteoclasts and macrophages were infected with DV, and viral antigen NS3 (*red color*) was detected by immunofluorescence staining at 24 h postinfection. Cells were countered stained with Hoechst (*blue*) and phalloidin (*green*). *Scale bar*, 200 μm. **d** Viral titers in culture supernatants of human osteoclasts and macrophages were determined by plaque assay. **e** TNF-α secretion from DV-infected human osteoclasts (2 × 10^4^/well) and macrophages (2 × 10^4^/well) were determined by ELISA. **f** DV-induced secretion of TNF-α and IL-6 from human osteoclasts were inhibited by anti-CLEC5A mAb (clone: 3E12A2, 1 μg in the 200 μl/well). Supernatants were harvested at 24 h postinfection to determine cytokine levels by ELISA. Data were collected and expressed as mean ± SEM from at least three independent experiments. For **f**, ANOVA tests were performed and all the infections were performed at M.O.I. = 5

To understand whether osteoclasts are susceptible to flaviviral infection, human CD14^+^ monocytes and mouse bone marrow cells (from STAT1-deficient mice) were incubated with M-CSF and RANKL for 17 and 9 days, respectively, to differentiate into osteoclasts. The success of osteoclast differentiation was confirmed by the presence of osteoclast marker TRAP, an alkaline phosphatase abundant in the osteoclast is able to hydrolyze the naphthol AS-BI phosphoric acid to couple with fast garnet GBC and form insoluble maroon dye deposited at sites of activity. TRAP^+^ giant cells were multinucleated (upper, Fig. 1a) and have ring-like F-actin structure by Hoechst/phalloidin double staining (lower, Fig. 1a). Similar finding was observed in mouse osteoclasts derived from bone marrow (Fig. S1a, b) as that of human osteoclasts. In addition, human osteoclasts expressed higher level of CD51/CD61 (left middle, Fig. 1b), while the expression level of CLEC5A in human osteoclasts was similar to macrophages (lower, Fig. 1b). To understand whether human osteoclasts were susceptible to DV infection, cells were incubated with DV (M.O.I. = 5) for 2 h, followed by washing to remove unbound virus. As shown in Fig. 1c, DV nonstructural protein NS3 (red color) was detectable in 7–10 % of osteoclasts at 24 h post-DV infection. The STAT1-deficent mouse osteoclasts and macrophages were also susceptible to DV infection, though the infectivity (2–5 %) is lower than human counterparts (Fig. S1c). To further confirm the replication of DV in human osteoclasts, we collected the supernatants from DV-infected osteoclasts and macrophages (M.O.I. = 5) to determine virus titer at different time points after incubation. We found that virus titers (Fig. 1d) and TNF-α secretion (Fig. 1e) from DV-infected osteoclasts and macrophages were similar. Moreover, anti-CLEC5A mAb was able to suppress the release of DV-induced cytokines (TNF-α and IL-6) from osteoclasts (Fig. 1f). All the observations suggest that DV not only infects and replicates in osteoclasts but also induces proinflammatory cytokine release from osteoclasts via CLEC5A.

### Osteoclasts are susceptible to flaviviral infections

**Fig. 2:**
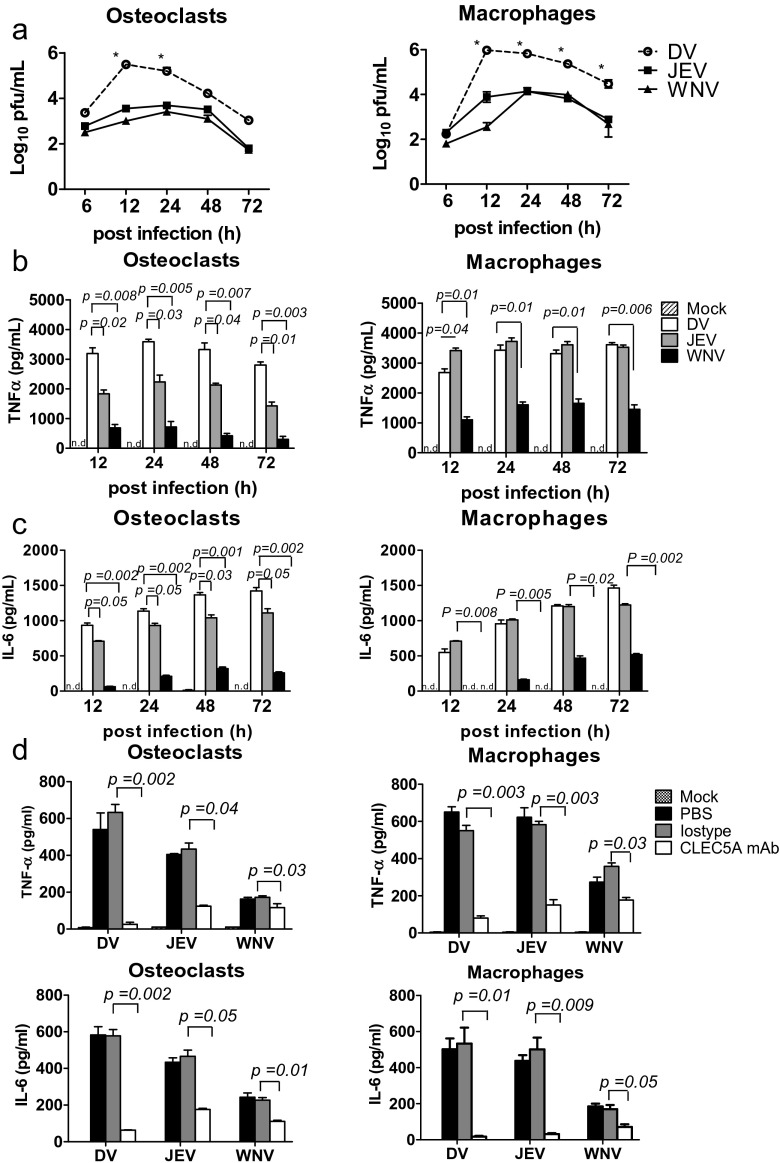
Osteoclasts are preferentially infected by dengue virus. **a–c** Human osteoclasts (7 × 10^5^/well) and macrophages (7 × 10^5^/well) were infected with DV, JEV, and WNV (M.O.I. = 5), respectively, and supernatants were harvested at indicated time points to determine virus titer by plaque assay (**a**), and supernatants were harvested to determine the release of TNF-α (**b**) and IL-6 (**c**) by ELISA. **d** Human osteoclasts (3 × 10^5^/well) and macrophages (3 × 10^5^/well) were preincubated with anti-CLEC5A mAb or isotype control, followed by DV, JEV, or WNV infection (M.O.I. = 5). Supernatants were harvested at 24 h postinfection to determine cytokine levels by ELISA. Data were collected and expressed as mean ± SEM from at least three independent experiments. ANOVA tests were performed. *P < 0.05, for DV group versus group of JEV and WNV infection

We further compared the susceptibility of human osteoclasts and macrophages to members of flaviviruses, including DV, Japanese encephalitis virus (JEV), and West Nile Virus (WNV). To address this question, cells were incubated with DV, JEV, and WNV, respectively (M.O.I. = 5), and supernatants were harvested to determine virus titer and cytokine release at different time points postinfection. We found that the DV titer was much higher than that of JEV and WNV, respectively, in the supernatants of both macrophages and osteoclasts (Fig. 2a). In addition, DV induced similar amount of TNF-α (Fig. 2b) and IL-6 (Fig. 2c) in human osteoclasts and macrophages, while JEV and WNV were less efficient to induce cytokine release from osteoclasts (left, Fig. 2b, c) than macrophage (right, Fig. 2b, c) under the same condition (left, Fig. 2b, c), despite JEV was as efficient as DV to induce the release of TNF-α and IL-6 from macrophages (right, Fig. 2b, c). This observation suggests that osteoclasts are preferentially susceptible to DV infection. In addition, antagonistic anti-CLEC5A mAb efficiently inhibited the secretion of TNF-α and IL-6 from human osteoclasts and macrophages (Fig. 2d). Thus, human osteoclasts are as susceptible as macrophages, and produce similar amounts of TNF-α and IL-6 after DV infection. Similar findings were observed in the mouse counterparts (Fig. S2). The *stat1*^*−/−*^ osteoclasts produced higher amount of TNF-α (left, Fig. S2a) and were more susceptible to DV than to JEV and WNV infection (left, Fig. S2b), while the susceptibility of *stat1*^*−/−*^ macrophages to all the three viruses were similar (right, Fig. S2a, b). The WT osteoclasts and macrophages produced less cytokines (Fig. S2a) and were more resistant to DV infection (100-fold less) than *stat1*^*−/−*^ osteoclasts and macrophages (Fig. S2b) under the same condition. Compared to STAT1-deficient cells (*stat1*^*−/−*^*clec5*^*+/+*^*)*, *stat1*^*−/−*^*clec5a*^*−/−*^ osteoclasts, and macrophages produced much less cytokine (Fig. S2a). This observation further suggests that DV-induced cytokine production is via CLEC5A.

### DV activates osteoclasts and upregulates osteolytic activity via CLEC5A

**Fig. 3:**
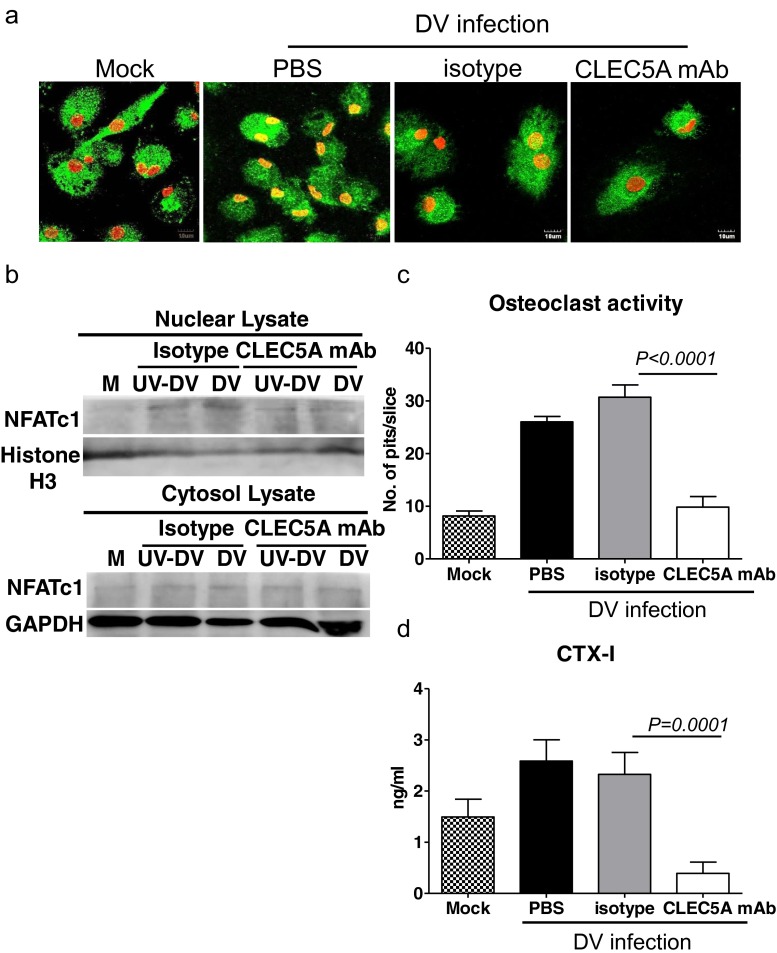
DV activates osteoclasts and upregulates osteolytic activity via CLEC5A. **a** Human osteoclasts (1 × 10^5^/well) were preincubated with anti-CLEC5A mAb or isotype control, followed by DV infection. Cells were fixed at 24 h postinfection for immunofluorescence staining and were examined under a confocal microscope (Olympus FV1000). *Scale bars*, 10 μm. *Green color*: NFATc1; *red color*: Hoechst 33342; *yellow color*: merge of NFATc1 (*green*) and Hoechst 33342 (*red*). **b** Human osteoclasts (5 × 10^6^) were preincubated with anti-CLEC5A mAb or isotype control, followed by DV infection. Cells were harvested at 12 h postinfection for WB analysis. **c** Determination of osteolytic activity by “pit formation” assay. Mature human osteoclasts were seeded on the bone slices and incubated with DV for 2 h in the presence of isotype control or CLEC5A mAb. The resorbed areas (pits) were examined under a microscope (Nikon) at day 6 after DV infection. Five images per each bone slice were randomly photographed, and the resorbed areas were counted and represented as mean ± SEM (under each picture) from three independent experiments. **d** Supernatants from bone slices incubated with human osteoclasts and DV were harvested at day 6 after DV infection to determine C-telopeptide of type I collagen (CTX-1) level by ELISA (CrossLaps for Culture). Data were collected and expressed as mean ± SEM ANOVA tests were performed and all the infections were performed at M.O.I. = 5

It has been shown that stimulation of CLEC5A by agonistic mAb results in augmented osteoclastogenesis in vitro [9], and crosslinking of CLEC5A-DAP12 induces intracellular calcium mobilization [17]. Thus, we asked whether DV was able to activate transcription factor NFATc1, a master regulator of osteoclast differentiation and activation, [18, 19] to upregulate osteolytic activity. To address this question, human osteoclasts were incubated with DV (M.O.I. = 5) for 24 h, followed by immunofluorescence staining to detect nuclear translocation of NFATc1. We found that NFATc1 located in the cytosol (green color, Fig. 3a) without DV infection (mock control). After DV infection, NFATc1 was translocated from cytosol to nuclei in cells treated with isotype antibody (yellow color). In contrast, anti-CLEC5A mAb effectively inhibited DV-mediated NFATc1 translocation. To further confirm this observation, we detect the presence of NFATc1 in nuclear lysate by western blot (Fig. 3b). At 12 h after infection, NFATc1 was detected in nuclear lysates when osteoclasts were infected with live or UV-inactivated DV, suggesting that NFATc1 translocation is independent of DV replication. Similarly, anti-CLEC5A mAb effectively inhibited DV-mediated NFATc1 translocation. This observation suggests that DV-induced NFATc1 translocation is via CLEC5A and is independent of DV replication.

We further examined the osteolytic activity of DV-infected osteoclasts by pit formation assay [20]. To address this question, osteoclasts (2 × 10^4^) were incubated with DV (M.O.I. = 5), in the presence or absence of anti-CLEC5A mAb for 2 h, followed by removal of unbound DV. The DV-infected osteoclasts were further incubated with bone slice in vitro for 6 days. Bone slice were then stained with toluidine blue to determine the resorbed area (pits) under an inverted microscope (Fig. 3c), and the supernatants were harvested to determine the amount of C-telopeptide of type I collagen (CTX-1), which was released from bone slice and regarded as the marker for bone turnover, by ELISA (Fig. 3d). The average pit numbers on bone slice were 8 ± 1 when incubated with uninfected osteoclasts. In contrast, DV infection upregulated osteolytic activity to increase pit numbers up to 27 ± 2 (Fig. 3c). Moreover, anti-CLEC5A mAb efficiently suppressed osteolytic activity in DV-infected osteoclasts, while isotype control antibody has no inhibitory effect (Fig. 3c). This observation further suggests that DV upregulates osteolytic activity via CLEC5A.

We further measured the amount of C-telopeptide of type I collagen (CTX-1) in the culture supernatant of osteoclasts before and after DV infection by ELISA. As shown in Fig. 3c, increased CTX-1 level (from 1.4 ± 0.17 to 2.59 ± 1.1 ng/ml) was observed after DV infection, while anti-CLEC5A mAb reduced CTX-1 release from bone slice (0.39 ± 0.24 ng/ml) (Fig. 3d). All the above observation suggested that DV is able to infect osteoclasts to increase osteolytic activity via CLEC5A.

### Dynamics of bone homeostasis after DV infection in vivo

**Fig. 4:**
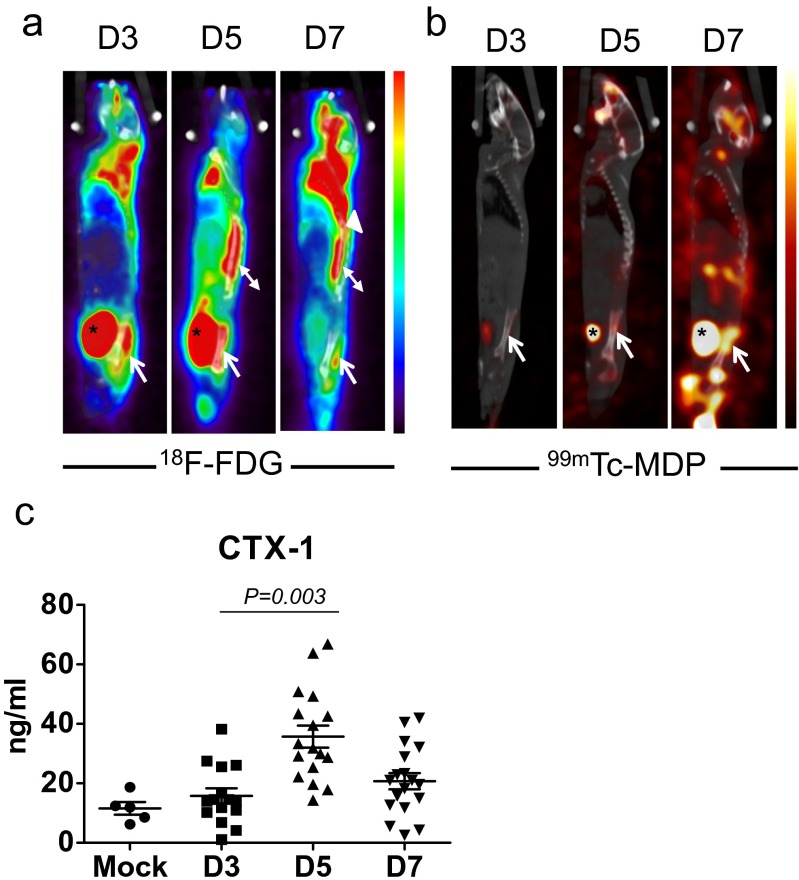
DV infection causes bone tissue inflammation and disturbance of bone homeostasis in STAT1-deficient mice. **a** Representative ^18^F-FDG PET/CT imaging of the STAT1-deficient mice at days 3, 5, and 7 after DV infection. Increased ^18^F-FDG uptake in the left sacroiliac junction (*arrow*) was observed from day 3 postinfection and subsided at day 7. Normal variations of tracer uptake in brown fat of the upper back region (*arrow head*) and paraspinal muscles (*double arrow head*) and tracer accumulation in the urinary bladder (*asterisk*) were noted. **b** ^99m^Tc-MDP SPECT/CT bone scan at day 5 postinfection showed slightly increased bony uptake of radiotracer in the upper part of left sacroiliac junction (*arrow*), and more intense uptake was detected at day 7. Tracer was accumulated in the urinary bladder (*asterisk*). Data from **a**, **b** were collected from five independent experiments. **c** Serum of DV-infected mice were harvested at indicated days after DV inoculation and C-telopeptide of type I collagen (CTX-1) were determined by RatLaps ELISA kit. Data were collected and expressed as mean ± SEM. ANOVA tests were performed

We further investigated whether DV infection caused acute bone inflammation in vivo. Because mice are not the natural host for DV, and wild-type mice are resistant to DV infection, the mouse-adapted DV (NGC-N strain) was used to infect the STAT1-deficiency mice as described previously [4] to investigate whether DV alters bone homeostasis in vivo. To address this question, STAT1-deficient mice were infected with DV (2 × 10^5^ PFUs/mouse) and examined by ^18^F-fluorodeoxyglucose-positron emission tomography/computed tomography (^18^F-FDG PET/CT) and technetium-99m MDP single-photon emission tomography/computed tomography (^99m^Tc-MDP SPECT/CT), to determine the inflammation status and relevant bone reaction, respectively. During the process of infection, activated granulocytes and lymphocytes had high glucose utilization; thus, regions of infection and inflammation were imaged as foci of high ^18^F-FDG uptake. It was interesting to note that increased glucose metabolic activity in the sacroiliac junction (arrows) was observed at day 3 postinfection (Fig. 4a). The glucose metabolic activity in sacroiliac junction peaked at day 5 and subsided subsequently at day 7 postinfection. This observation suggests that DV infection caused severe inflammatory reaction in bone and surrounding soft tissues. Furthermore, uptake of ^99m^Tc-MDP gradually increased in the sacroiliac junction (arrows) from day 3 and reached peak at day 7 postinfection (Fig. 4b). This observation indicated that the ^99m^Tc-MDP delivered by blood flow was incorporated into the osteogenic new bone formation incited by the infection. Thus, the metabolic imaging not only disclosed active inflammation involving the sacroiliac junction at early infection but also detected the reparative process which occurred later than the acute inflammation. We further detected the serum level of C-telopeptide of type I collagen (CTX-1) released from type I collagen in bone tissue. The basal CTX-1 serum level was 12 ng/ml before infection. After infection, CTX-1 serum level increased from day 3, peaked at day 5, and subsided at day 7 post-DV infection (Fig. 4c). The trend of CTX-1 serum level was in accord with the ^18^F-FDG uptake in STAT1-deficient mice (Fig. 4a), suggesting that DV induced remarkable inflammation at day 5 postinfection, followed by increased osteolytic activity and bony destruction before reactive new bone formation (as revealed by ^99m^Tc-MDP uptake in Fig. 4b).

### CLEC5A-deficient mice resist DV-induced disturbance of bone homeostasis

**Fig. 5:**
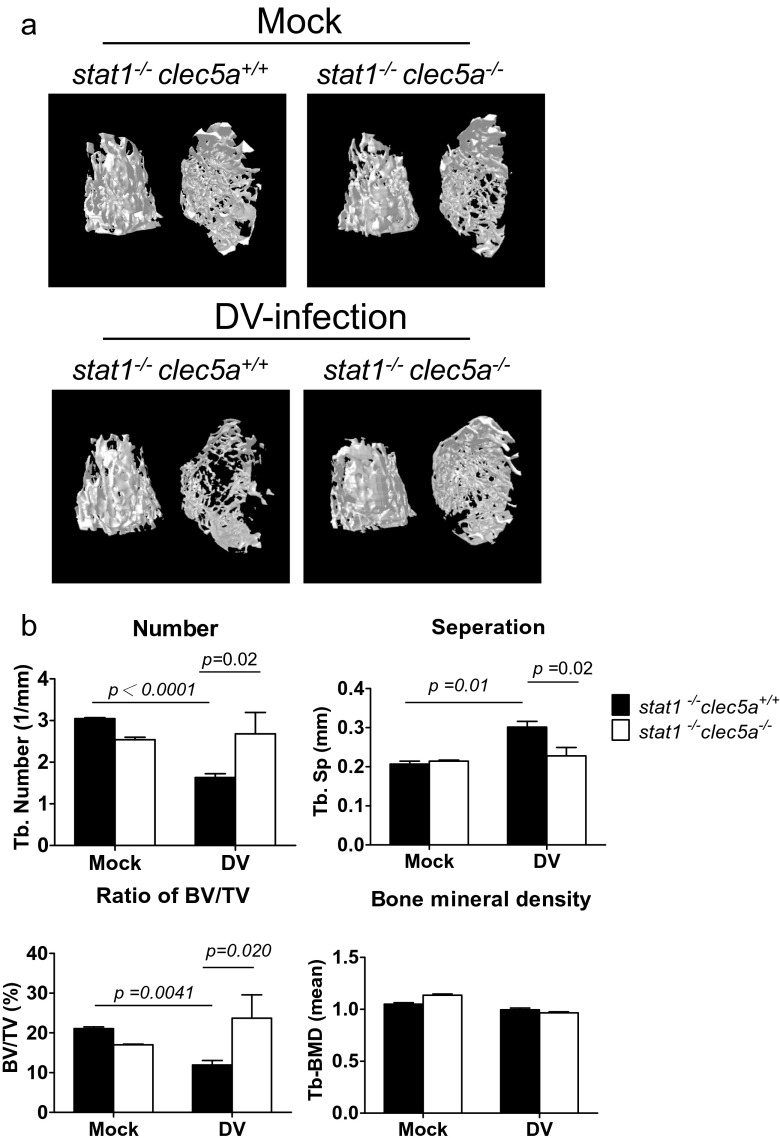
CLEC5A is responsible for DV-induced dynamic change of bone structure in vivo. **a** The imaging of three-dimensional microcomputed tomography for trabecular bones from DV-challenged *stat1* ^*−/−*^ *clec5a* ^*+/+*^ and *stat1* ^*−/−*^ *clec5* ^*−/−*^ mice (2 × 10^5^ PFUs per mouse) at day 7 postinfection. **b** Parameters for the trabecular bone number (Tb-N), bone volume to tissue volume (BV/TV), separation between trabeculae distance (Tb-Sp), and bone mineral density of trabecular bone were quantitated before and after DV infection. Data were collected and expressed as mean ± SEM from at five independent experiments. Two-tailed Student’s *t* tests were performed

To understand the role of CLEC5A in DV-induced disturbance of bone homeostasis in vivo, STAT1-deficient mice (wild-type mice are resistant to DV infection) were crossed with CLEC5A-deficient mice to produce *stat1*^*−/−*^*clec5a*^*−/−*^ double knockout mice as described previously [5]. At day 5 postinfection, DV replicated efficiently in bone marrow of STAT1-deficient (*stat1*^*−/−*^*clec5a*^*+/+*^) mice (Fig. S3a) and upregulation of *tnf-*α (Fig. S3b) and *il-6* (Fig. S3c) was also noted in the *stat1*^*−/−*^*clec5a*^*+/+*^ mice, but not *stat1*^*−/−*^*clec5a*^*−/−*^ mice. We further compared the dynamics of bone homeostasis by the three-dimensional microcomputed tomography (Fig. 5a). As shown in Fig. 5b, DV infection caused the decrease of trabecular bone number (Tb-N) (black column, upper left) and bone volume to tissue volume (BV/TV) (black column, lower left) at day 7 after DV infection. In contrast, the Tb-N and BV/TV ratio were higher in CLEC5A-deficinet mice (white column). Moreover, the separation between trabeculae distance (Tb-Sp) increased after DV infection, and the Tb-Sp in CLEC5A-deficient mice is maintained in the same level (upper right, Fig. 5b). However, the bone mineral density did not change after DV infection in both WT littermates and CLEC5A-deficinet mice (lower right, Fig. 5b). This observation suggests that CLEC5A regulates DV-induced disturbance of bone homeostasis.

### Anti-CLEC 5A mAb counteracts DV-induced disturbance of bone homeostasis

**Fig. 6:**
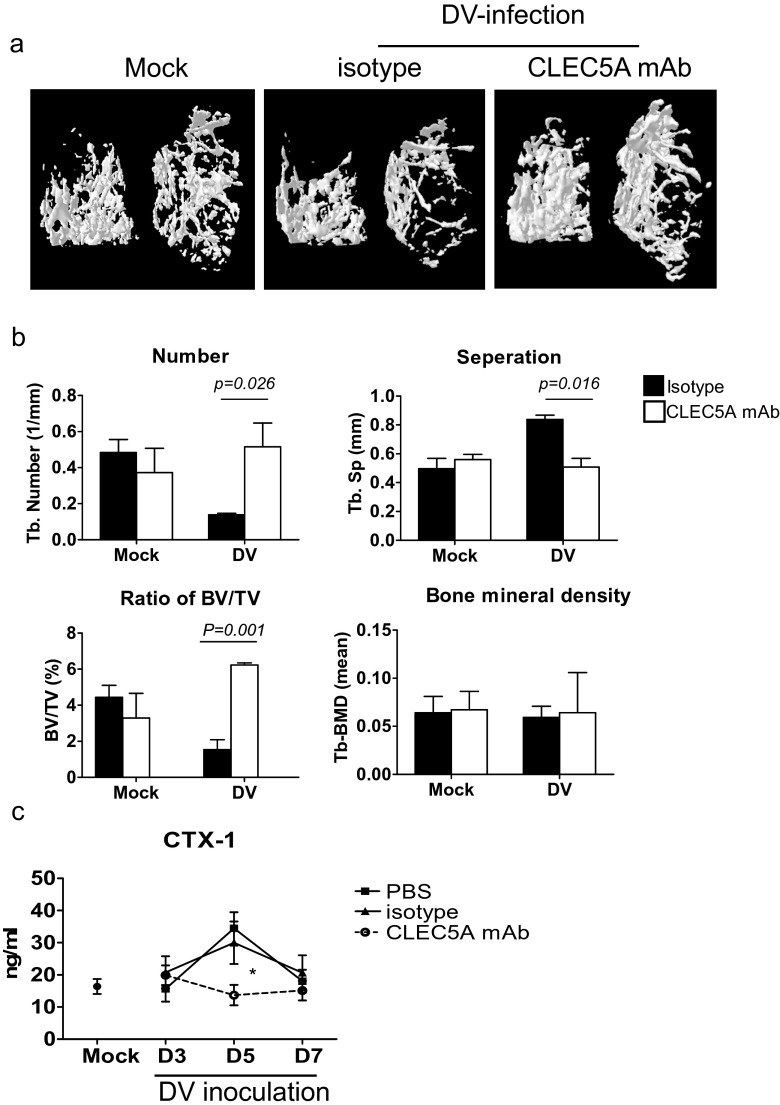
Anti-mCLEC5A mAb prevents DV-induced bone loss. **a** The imaging of three-dimensional microcomputed tomography for trabecular bone from DV-challenged STAT1-deficient mice (*stat1* ^*−/−*^ *clec5a* ^*+/+*^) administered with anti-CLEC5A mAb (clone: 3D2H6, 100 μg per mouse) or isotype control at day 7 postinfection. **b** Parameters for the trabecular bone number (Tb-N), bone volume to tissue volume (BV/TV), separation between trabeculae distance (Tb-Sp), and bone mineral density of trabecular bone were quantitated before and after DV infection. **c** Serum were harvested at indicated days after DV infection, and C-telopeptide of type I collagen (CTX-1) were determined by the RatLaps ELISA kit. Data were collected and expressed as mean ± SEM from five independent experiments. Two-tailed Student’s *t* tests were performed

We further asked whether administration of antagonistic anti-CLEC5A mAb [4] was able to modulate bone homeostasis in DV-infected WT mice (*stat1*^*−/−*^*clec5a*^*+/+*^). As shown in Fig. 6a, reduction of bone mass was noted in mice treated with isotype mAb at day 7 post-DV infection. In contrast, administration of anti-CLEC5A mAb efficiently inhibited DV-induced reduction of bone mass (right most, Fig. 6a). Furthermore, anti-CLEC5A mAb (while column, Fig. 6b) was able to prevent DV-induced reduction of trabecular bone number (upper left, Fig. 6b), BV/TV ratio (lower left, Fig. 6b), and the increase of trabeculae distance (upper right, Fig. 6b). However, anti-CLEC5A mAb did not alter bone mineral density in mice with or without DV infection (lower right, Fig. 6b).

Furthermore, anti-CLEC5A mAb efficiently prevented the increase of CTX-1 serum level after DV infection (Fig. 6c). All the above evidence suggests that DV-induced bone inflammation and disturbance of bone homeostasis is via CLEC5A, and blockade of CLEC5A is able to attenuate osteolytic activity in DV-infected mice. Thus, antagonistic anti-CLEC5A mAb is not only able to attenuate DV-induced lethality and neuronal inflammation [4–6] but also has the potential to suppress DV-induced osteolytic activity to maintain bone homeostasis during DV infection.

## Discussion

While macrophages and dendritic cells are susceptible to DV infection, the role of osteoclasts in DV infection has not been addressed. Here, we demonstrated that DV infected human osteoclasts and enhanced osteolytic activity via CLEC5A (Fig. 3). Moreover, the imaging data further demonstrated that DV caused inflammatory reactions in bone tissue (Fig. 4) and increased osteolytic activity in vivo (Figs. 5 and 6). All these observation suggests that DV can infect and upregulate osteolytic activity in vivo.

One of the typical clinical symptoms of dengue patients is the “shattering pain,” which cannot be suppressed by the nonsteroid anti-inflammatory drug (NSAID) in dengue patients. Thus, dengue infection is also known as “breakbone fever.” However, the underlying mechanism for the extremely painful sensation of muscle and joints observed in dengue patients is still unclear. It has been shown that increased osteolytic activity contributes to pain sensation in inflammatory diseases, and the normally innocuous stimuli produce pain sensation during the inflammatory status [21]. Moreover, the increased osteoclastic bone resorption are frequently associated with bone pain, which is likely due to the acidosis environment caused by the protons secreted from activated osteoclasts [13]. Furthermore, increased osteolytic activity does not only contribute to complete Freund’s adjuvant (CFA)-induced bone pain [13] but also contributes to the pain sensation in patients suffered from cancer bone pain [22], rheumatoid arthritis, and osteoporosis [23]. Therefore, it is reasonable to speculate that DV-induced inflammatory reaction in bone tissues, as well as the increased osteolytic activity of DV-infected osteoclasts, may contribute the severe pain sensation in dengue patients.

Up to now, only very few human viruses (such as measles virus and human immunodeficiency virus) have been reported to infect osteoclasts and modulate cell differentiation [24, 25]. However, there is no any report showing that virus can infect osteoclasts and upregulates its osteolytic activity. Results from this study clearly demonstrate that DV can infect and replicate in osteoclasts, as well as upregulates osteolytic activity to disturb bone homeostasis in mouse model. Thus, the painful sensation in dengue patients may be due to the unique ability of DV to activate osteoclasts and disturb bone homeostasis. This argument is supported by the observation that the CTX-1 serum level is also upregulated in dengue patients (Fig. S4), suggesting increased osteolytic activity during DV infection.

Because mice are not the natural host for DV and wild-type mice are resistant to DV infection, even though extremely high dose of DV (2 × 10^9^ pfu/mouse) did cause mild hemorrhaging at skin after intradermal injection in wild-type mice [26]. Thus, the immunodeficient mice AG129 strain (deficiency of type I and type II interferon receptors) [27] and STAT1-deficiency mice [4] infected by the mouse-adapted DV strains were commonly used to investigate the pathogenesis of DV infection in vivo, when nonhuman primates are not available. Because interferons play critical roles to inhibit DV infection, thus AG129 mice were highly susceptible to DV infection and were developed for vaccine testing because sustained DV replication were noted after DV inoculation [28]. Similarly, STAT1 is a key regulator located at the downstream of IFN signaling cascade; thus, STAT1-deficiency mice is also susceptible to DV infection. Harris’s group demonstrated that AG129 mice bear prolonged viral loads in the serum and tissues than that in the STAT1-deficient mice after inoculation of clinical isolation of DENV2 (10^8^ pfu, PL046/mouse) to AG129 and STAT1. Moreover, deficiency of IFNα/β and IFNγ receptors, but not STAT1, reduced the numbers of activated NK and B cells after DENV infection [29]. Therefore, Harris’s group suggested that IFNR-dependent control of primary DEN infection involves both STAT1-dependent and STAT1-independent mechanisms. The STAT1 pathway is necessary for clearing the initial viral load, whereas the STAT1-independent pathway controls later viral burden and prevents DEN disease in mice. Thus, STAT1-deficient mice still preserves partial host immunity against DV infection. In this study, we used the mouse-adapted NGC strain to infect Stat1-deficient mice for in vivo study as described previously [4]. Even though the dynamic change of bone homeostasis was observed in the STAT1-deficient mice after DV infection, this phenomenon needs to be further confirmed in nonhuman primates or DV-infected patients in the future.

In addition to DV, the arthritogenic alphaviruses (including Rosa River virus (RRV) and chikungunya virus (CHIKV)) have been shown to induce join pain and polyarthralgias, and bone lesions in joints were found in CHIKV-infected patients [30]. The molecular mechanism is unknown until Chen et al. showed that RRV-infected osteoblasts produce high levels of IL-6 and RANKL, while the level of osteoprotegerin (OPG), the decoy receptor of RANKL, was downregulated. The increased RANKL/OPG ratio contributed to upregulation of osteoclast activity and bone loss in RRV-infected mice, while anti-IL-6 neutralizing mAb was able to block RRV-induced bone loss [31]. In contrast to alphaviruses, OPG is upregulated dramatically after DV infection [32]. They further demonstrated that OPG bound to von Willebrand factor (VWF) to prevent platelet interaction and regulate host immunity via neutralizing TRAIL and RANKL. Therefore, RRV and DV apparently use distinct mechanism to upregulate osteoclast activity to perturb bone homeostasis.

Our data further support the important roles of CLEC5A in the pathogenesis of flaviviral infection. We have shown that CLEC5A is critical in DV-induced hemorrhaging shock and NALP3 inflammasome activation, and blockade of CLEC5A by antagonistic anti-CLEC5A mAb inhibits DV- and JEV-induced lethality [4–6]. This argument is supported by the observation that the movement and activity of anti-CLEC5A mAb-treated *stat1*^*−/−*^*clec5a*^*+/+*^ mice were not hampered by DV infection, and administration of anti-CLEC5A mAb was able to prevent DV-induced paralyzed (Supplementary data, video tape). Thus, blockade of CLEC5A may become a novel strategy to relieve the severe pain sensation of dengue patients in the future.

ESM 2The 6 to 8 week old *stat1* ^*−/−*^ */clec5a* ^*+/+*^ were inoculated with New Guinea C-N strain (2 × 10^5^ PFUs per mouse) and their activity was observed and taped at day 7 after infection. While isotype immunoglobulin-treated mice were paralyzed after DV infection, the movement capability of anti-mCLEC5A mAb-treated mice was similar to mock-infected mice. (MP4 12556 kb)

## Electronic supplementary material

Below is the link to the electronic supplementary material.

ESM 1 (PDF 202 kb)
