# Supplementary material for: CLEC5A is critical for dengue virus-induced osteoclast activation and bone homeostasis
Source: J Mol Med (Berl). 2016 Mar 31;94:1025–37. doi: 10.1007/s00109-016-1409-0 (PMC4992505; doi:10.1007/s00109-016-1409-0)
Supplement: Supplementary file 2 — (PDF 202 kb) [file 109_2016_1409_MOESM1_ESM.pdf]

Electronic supplementary figures and legends

CLEC5A is Critical for Dengue Virus-induced Osteoclast Activation and Bone Homeostasis

Ya-Lang Huang,[¶] Szu-Ting Chen, Ren-Shyan Liu, Yen-Hsu Chen, Chun-Yu Lin, Chung-Hao Huang, Pei-Yuen Su, Ching-Len Liao, Shie-Liang Hsieh

Journal of Molecular Medicine 2016

Supplementary Video

The 6 to 8 week old *stat1*$^{-/-}$/*clec5a*$^{+/+}$ were inoculated with New Guinea C-N strain (2×10^5 PFUs per mouse) and their activity was observed and taped at day 7 after infection. While isotype immunoglobulin-treated mice were paralyzed after DV infection, the movement capability of anti-mCLEC5A mAb-treated mice was similar to mock-infected mice.

Fig. S1

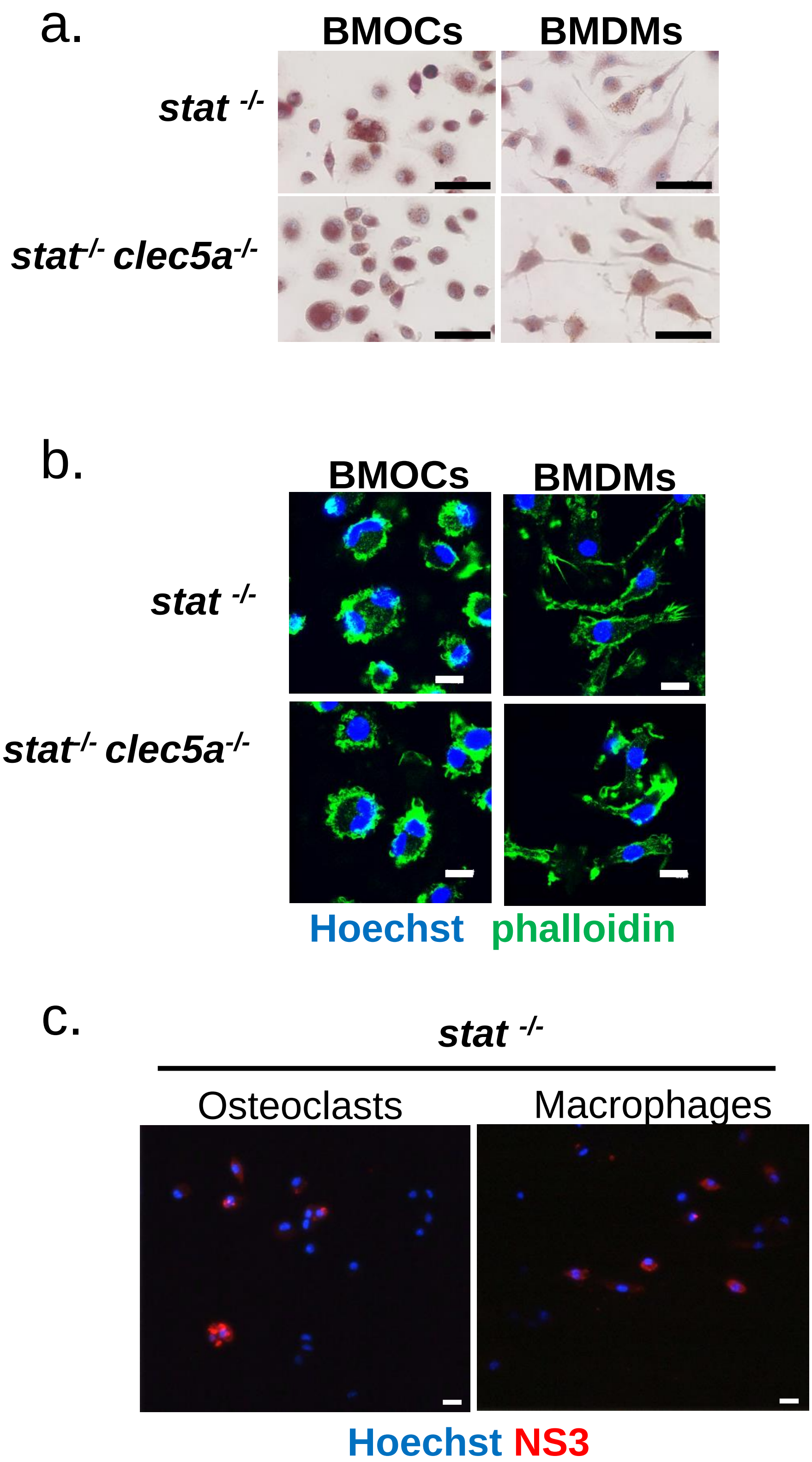

Fig. S1 Morphology of mouse bone marrow derived-osteoclasts and macrophages. Bone marrows isolated form *stat1*$^{-/-}$ and *stat1*$^{-/-}$ *clec5a*$^{-/-}$ mice were differentiated into osteoclasts and macrophages, respectively. Cells were performed **a**) TRAP assay and **b**) immunofluorescence assay with phalloidin (green) and Hoechst (blue) to confirm their identity. Scale bar, 40 µm. **c)** Osteoclasts and macrophages were infected with DV2, followed by detecting DV replication using antibody against viral antigen NS3 (red color) at 48 h post infection. Cells were countered stained with Hoechst (blue). Scale bar, 40 µm

Fig. S2

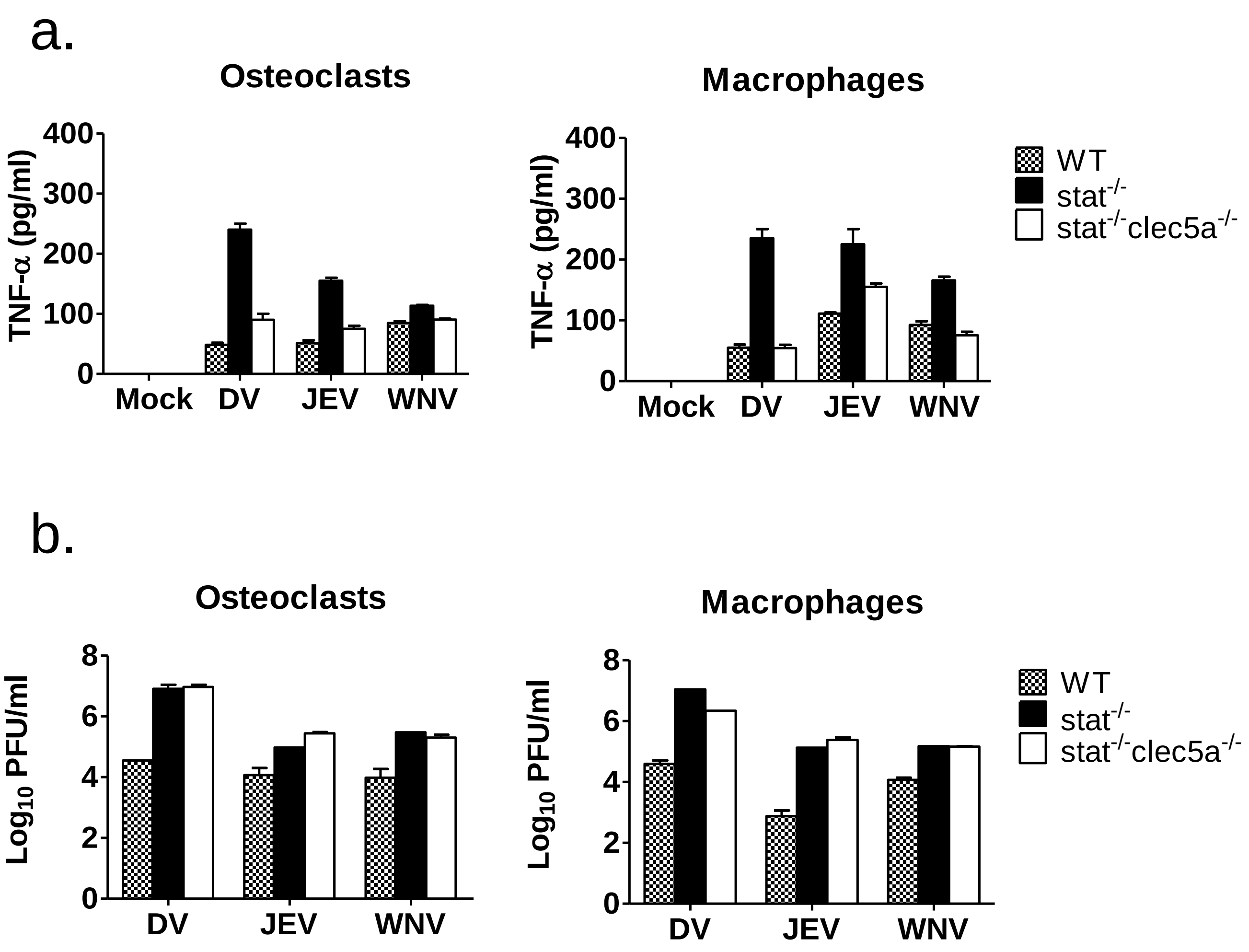

Fig. S2 Differential susceptibility and cytokine production in flavivirus-infected mouse osteoclasts and macrophages.
Mouse osteoclasts ($3x10^5$/well) and macrophages ($3x10^5$/well) were infected with DV2, JEV and WNV (M.O.I. = 10) respectively, and supernatants were harvested at 24 h post infection to determine virus titer by plaque assay, while the cytokine levels were determined by ELISA.

Fig. S3

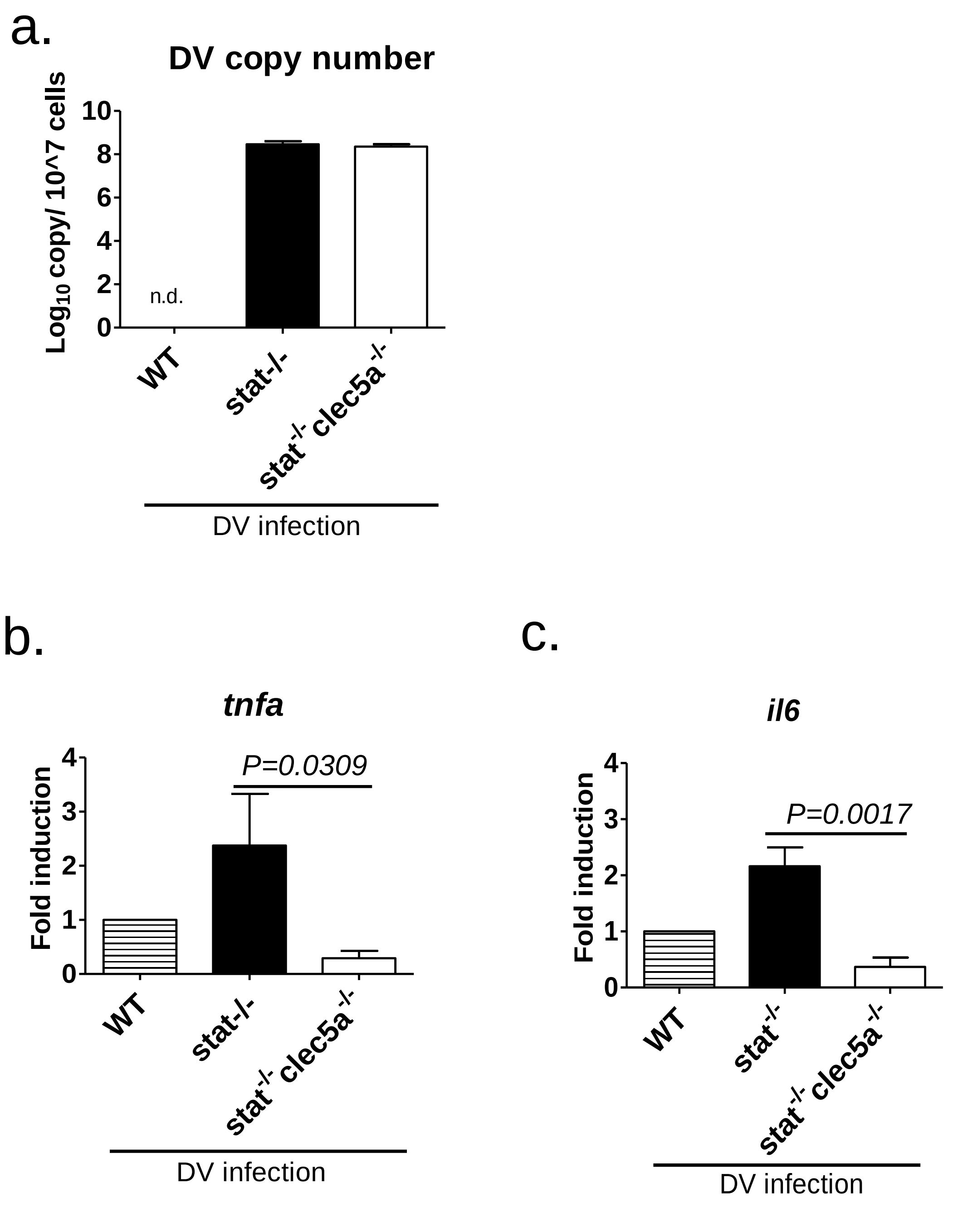

Fig. S3 Dengue virus replicates in bone marrow tissue and induces proinflammatory cytokine secretion in vivo.
Mice were intraperitoneally inoculated with DEN2V (NGC-N, 2×10^5 PFUs/mouse), and bone marrow cells were harvested at day 5 post infection to detect **a)** viral copy numbers and the expression of **b**) *tnf* and **c**) *il-6* by qRT-PCR. Data were collected and expressed as mean ± s.e.m. from five independent experiments. Two-tailed Student's *t*-tests were performed.

Fig. S4

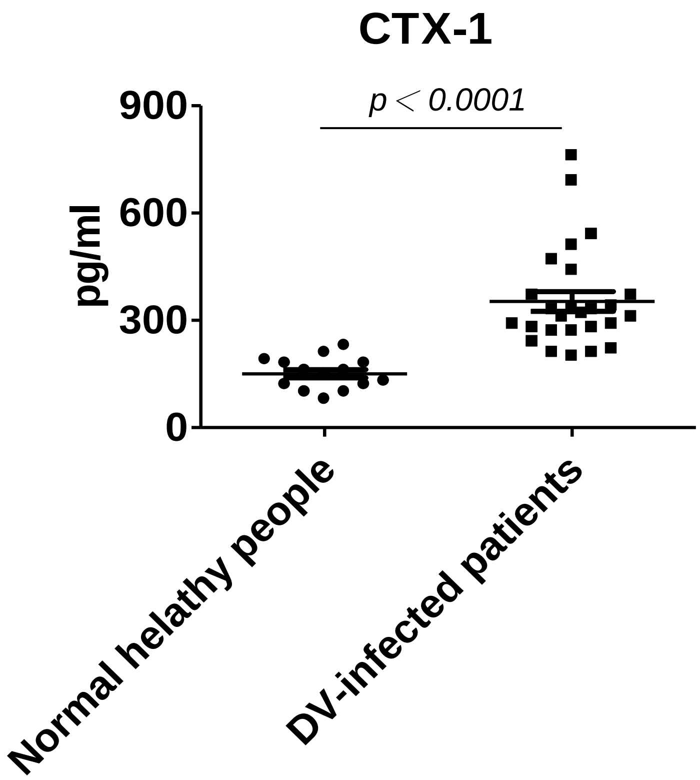

Fig. S3 Dengue virus replicates in bone marrow tissue and induces proinflammatory cytokine secretion in vivo.
Mice were intraperitoneally inoculated with DEN2V (NGC-N, 2×10^5 PFUs/mouse), and bone marrow cells were harvested at day 5 post infection to detect **a)** viral copy numbers and the expression of **b**) *tnf* and **c**) *il-6* by qRT-PCR. Data were collected and expressed as mean ± s.e.m. from five independent experiments. Two-tailed Student’s *t*-tests were performed.
